# Stem Cells Have Different Needs for REST

**DOI:** 10.1371/journal.pbio.0060271

**Published:** 2008-10-28

**Authors:** Ola Hermanson

## Abstract

REST is a well known repressor of neuronal gene expression. Genome-wide analysis of REST occupancy in different cell types now reveals new and cell-specific roles for REST in embryonic stem cells.

Embryonic stem cells (ESCs) are believed to possess the innate capacity to differentiate into any of the multitude of cell types that make up the body, a capacity known as pluripotency (http://en.wikipedia.org/wiki/Pluripotent). For a cell to differentiate and adopt the identity of a specific cell lineage, transcriptional mechanisms must “switch on” a suite of genes that encode proteins characteristic of that cell type. Simultaneously, other genes characteristic for other cell types must reliably be “switched off.” As the proper regulation of gene expression is essential for cellular differentiation and normal development, understanding how differentiation programs regulate the correct number and types of cells in a developing organism is a fundamental issue in biology and medicine.

Differentiation programs employ regulatory factors that both promote and repress transcription. For example, certain vertebrate activating transcription factors (http://en.wikipedia.org/wiki/Transcription_(genetics)), including Mash1 (Mammalian achaete scute-like 1), Math1 (Mouse atonal homologue 1), and the Neurogenin family [1], have been shown to be sufficient and/or required to promote differentiation into a neuronal cell type, while transcriptional repressors have been shown to play a key role in determining pluripotency and differentiation [2]. The Notch family of membrane receptors exerts strong inhibition of differentiation into neurons by increasing the cellular levels of powerful transcriptional repressors, such as the nuclear factors Hes1–5. Most factors of this family function as repressors of neurogenesis (http://en.wikipedia.org/wiki/Neurogenesis) by directly binding to the promoter of neurogenic genes such as Mash1, repressing the gene [3].

## REST Is a Transcriptional Repressor of Neuronal Genes

Although Notch signaling and Hes activity have been shown to be fundamental in repressing neuronal differentiation during early embryogenesis, it remained at first unclear how repression of neuronal gene expression was maintained in non-neural cells—permanently throughout the life of a differentiated non-neural cell. After the identification of a 21– to 23–base pair silencing element named Repressor Element 1 (RE1) in the promoter of certain neuronal genes, in 1995 Gail Mandel's and David Anderson's labs independently reported the discovery of a transcriptional repressor binding to this element, which they named REST (RE1 silencing transcription factor) and NRSF (neuron-restrictive silencing factor), respectively (from here referred to as REST) [4,5]. REST turned out to be a 116-kD zinc finger protein binding to the classical RE1 and containing two repressor domains in the N- and C-terminal domains, respectively.

**Figure 1:**
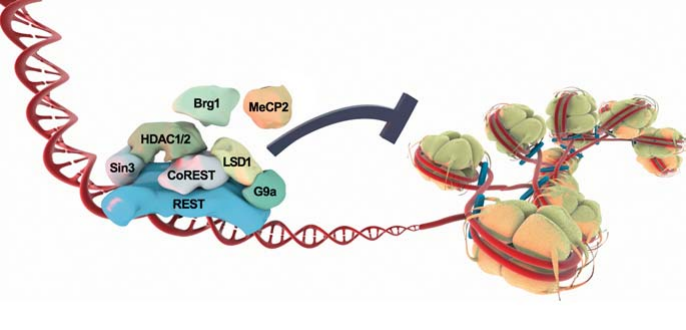
REST Repression Is Associated with Many Different Transcriptional Coregulators A schematic model of REST interactions with some selected transcriptional coregulators and chromatin modifying proteins. HDAC1/2 deacetylate lysine 9 on histone H3 (H3K9), and low acetylation and high methylation of this lysine when situated in a promoter close to a transcription start site is associated with transcriptional repression. The histone demethylase LSD1 represses transcription by demethylating lysine 4 on histone H3 (H3K4). H3K4 is a residue that, when (tri)methylated, attracts and recruits transcription initiation factors, and thus H3K4 methylation is associated with transcriptional activation, in contrast to, e.g., H3K9.

Since its discovery, REST has been the subject of intense research in the fields of developmental biology and transcription. Due to the well-defined RE1 response element and the subsequent identification of numerous endogenous target genes containing this site [6,7], REST proved to be versatile for investigating basic transcriptional mechanisms including more epigenetic mechanisms involving activation or silencing by modification of chromatin proteins (http://en.wikipedia.org/wiki/Chromatin), histones associated with DNA. Soon after the initial reports connecting transcriptional activation with histone acetylation (associated with “open” chromatin and accessible DNA) and transcriptional repression with histone deacetylation (associated with “compact” chromatin and less accessible DNA), the N-terminal repression domain of REST was shown to bind the transcriptional repressor Sin3A and associated histone deacetylases (HDACs) such as HDAC1 and HDAC2 (Figure 1) [8,9].

Interestingly, the C-terminal domain of REST was shown to interact with a distinct corepressor named CoREST that formed a specific protein complex that used different and previously uncharacterized strategies to modify chromatin and thereby repress neuronal genes [8,9]. While isolating a protein complex originally associated with a corepressor unrelated to REST named CtBP, Shi and colleagues found this complex to consist of a number of factors associated with REST, including CoREST, HDAC1/2, the histone methyl transferase G9a, and the histone demethylase LSD1 (Figure 1) [10–12]. These discoveries of REST- and CoREST-interacting enzymes with specific chromatin-modifying abilities served to reveal how complex the regulation of repression is, and suggested that the mechanisms of REST repression of neuronal genes were more sophisticated than first assumed. The picture has gotten even more complicated as REST has been shown to interact with many additional transcriptional regulators, such as the basal transcription factor TATA-binding protein (TBP) and the chromatin-remodeling factors BRG1 and Baf57 [9].

In association with the role for REST in repressing neuronal gene expression, it has also been implicated in neurological disorders. REST has been shown to regulate the neurotrophic factor BDNF (brain-derived neurotrophic factor) with implications for psychiatric disease. By interactions with various transcription factors, REST has also been associated with the neurodevelopmental disorder (http://en.wikipedia.org/wiki/Neurodevelopmental_disorder) Rett syndrome, Huntington disease, and recently with X-linked mental retardation [9,13,14].

## Additional Roles for REST in Other Cellular Events

**Figure 2:**
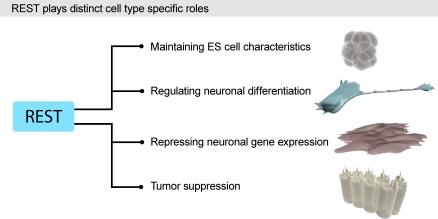
A Schematic Summary of Some Cell Type–Specific Roles for REST Recent reports show a novel role for REST in maintaining ESC phenotype by being a close part of the core transcriptional network of Oct4/Sox2/Nanog in ESCs (see text). REST has previously been suggested to repress neuronal differentiation in NSCs and medulloblastoma cells, in addition to its well-established role in repressing neuronal genes in non-neural cells. REST has also been shown to act as a tumor suppressor by repressing epithelial cell transformation, a role that could be linked to the regulation of cell adhesion genes (see text).

The increased knowledge of the complex nature of the transcriptional regulation by REST has been accompanied by an increasingly more complicated view of the functional roles for REST in physiology and pathology (Figure 2). The perhaps most unexpected finding was the identification of REST as a tumor suppressor in an unbiased screen for suppressors of epithelial cell transformation and thus tumorigenesis [15]. REST had already previously been implicated in tumor biology as an oncogene (http://en.wikipedia.org/wiki/Oncogene) for its ability to repress neuronal genes and thereby presumably repress differentiation of neuroectodermal tumor cells [16].

Concurrently with the first reports of REST as a tumor suppressor, it was demonstrated that the levels of REST protein in combination with how and where REST binds to the genome are critical parameters for the regulation of physiological neuronal differentiation in neural stem cells (NSCs) and also in ESCs [17,18]. It had been known for some time that REST and alternatively spliced isoforms thereof are expressed in undifferentiated neural progenitors [19], but it now became evident that REST levels are high also in ESCs, where its presence was shown to be essential for keeping neuronal gene expression low [17]. Interestingly, similar to what was recently reported for REST in epithelial cells undergoing transformation [20], REST was shown to undergo post-translational degradation during the progression of neuronal differentiation, thereby relieving repression and ultimately allowing neuronal gene expression to occur [17,18].

Lately, it has been shown that REST may play additional roles in cellular events not necessarily associated with its repression of neuronal genes. Deletion of REST in ESCs was reported to result in loss of self-renewal and a markedly decreased expression of members of the transcriptional network required for the self-renewal of ESCs, i.e., Oct4, Sox2, and Nanog (see further discussion below), although any putative direct connection between REST and these factors was not noted [21]. It has also been shown that REST may bind to other binding sites than the “classical” REST element RE1 [22], and studies using genome-wide sequencing after chromatin immunoprecipitation (ChIP-Seq; http://en.wikipedia.org/wiki/Chip-Sequencing) in human T cells suggested that REST also interacted with gene networks involved in pancreatic islet ß cell development [23]. These observations suggest that REST may not only repress neuronal genes and differentiation, but may play a more general role in regulation of cell-characteristic gene expression.

## A Genome-Wide Look at REST in Embryonic and Neural Stem Cells

In this issue of *PLoS Biology*, Johnson, Teh and colleagues report significant progress in the understanding of cell-specific roles for transcription factors in general and REST in particular by genome-wide studies of REST occupancy in different cell types, with particular focus on ESCs [24]. To achieve their results, the authors have utilized many strategies, such as remarkably sensitive ChIP-Seq approaches and extensive bioinformatics. ChIP-Seq techniques are usually based on using an antibody that recognizes the antigen, in this case REST, to pull the protein down from cell extracts while it is still bound to DNA, subsequently digesting the DNA and reversing the binding with the factor. Mass-sequencing techniques are then used to identify the DNA sequences and thus the gene loci that have been pulled down with the antibody [25]. By using this and similar techniques, the authors demonstrate that REST occupies both common and distinct sites in different cell types when comparing results from ESCs, ESC-derived NSCs, and fibroblasts. When examining binding to the “classical” REST binding site, they found roughly half (44%) of these sites to be occupied in all three cell types, while others showed more cell-restrictive occupancy. When examining REST binding in a more unbiased approach, they made the important observation that while the sites occupied by REST in NSCs were mostly shared with ESCs, large sets of REST binding sites were unique for ESCs. It is well known that transcription factors and associated complexes are targeted by intracellular signaling factors, and that such regulation can influence the gene expression in a cell-specific manner [26]. This novel observation made by genome-wide comparisons of REST binding in different cell types expands this old view by providing essential insight into how the same transcription factor can bind to different genes in a cell-specific manner, and thereby exert similar and distinct effects in different cell lineages.

The next interesting finding was made when the authors analyzed the classes of genes regulated by REST with special emphasis on sites that showed enriched occupancy specifically in ESCs [24]. Whereas neuronal genes were still a major target group for REST when compared to the whole genome, the current analysis revealed novel important REST targets. Cell adhesion genes were found to be one such group of major targets, in partial accordance with previous reports [22,27]. Interestingly, a novel major group of genes targeted by REST preferentially in ESCs turned out to be signaling molecules associated with the Wnt pathways, including no less than around ten Wnts (including Wnt1 and Wnt3a) and the Wnt signaling associated factors Dishevelled 1–3 [24].

There are several reasons why this finding is noteworthy. Wnts constitute a major class of extracellular signal substances, and different Wnts affect specific intracellular signaling pathways with profound effects on cell proliferation, death, migration, polarity, progenitor expansion, differentiation, and maturation. Thus changes in Wnt signaling will induce a plethora of effects on several key aspects of the ESC phenotype. Moreover, this regulatory event provides a prime example of how modulation of a single transcription factor may have major secondary and tertiary bystander effects, with important implications for the specificity issue of drugs modulating transcriptional regulators.

Arguably, the most timely of the observations made by Johnson, Teh and colleagues is that REST is a direct part of the Oct4/Sox2/Nanog transcriptional network in ESCs and that many REST targets are other transcription factors that are also regulated by the Oct4/Sox2/Nanog factors in ESCs [24]. Oct4/Sox2/Nanog are transcription factors that constitute a “core” in a transcriptional network essential for the undifferentiated and pluripotent state of ESCs. These factors maintain the state of ESCs by regulating the expression levels of each other and additional essential transcriptional regulators [28]. As mentioned above, it has been reported that gene deletion of REST results in a decrease in the expression of Oct4/Sox2/Nanog [21], and further that REST is a target of Oct4/Sox2/Nanog [29], and may be an interacting part of the transcriptional network of Nanog [28]. The current report expands these findings by showing that REST shares a significant number of target genes with Oct4, Sox2, and Nanog, and that several of these REST targets are genes encoding for factors that are essential for ESC maintenance, including Nanog itself.

This study also raises many new questions. There are now several reports of the generation of induced pluripotent stem (iPS) cells achieved by introducing small sets of transcription factors such as Oct4, Sox2, and Nanog into differentiated cells, and it will be interesting to see whether regulation of REST is part of the dedifferentiation events. The very large amounts of data generated by the genome-wide studies by Johnson, The, et al. provide a vast dataset for deeper examination by the field, and it will be of immediate interest to study similarities and differences between epigenetic regulatory mechanisms at common and cell-specific REST sites. The cell-specific differences in REST occupancy serve to inspire further genome-wide investigations of the occupancy of other transcription factors as well as REST-associated chromatin modifying proteins in distinct cell types and different cellular contexts. Using these types of genome-wide studies, there is unlimited potential to shed new light on old problems concerning the influence of metabolic and environmental cues on physiological stem cell characteristics as well as pathological events such as tumorigenesis.

## Abbreviations

ChIP: chromatin immunoprecipitation
ESC: embryonic stem cell
HDAC: histone deacetylase
iPS: induced pluripotent stem
NSC: neural stem cell
RE1: Repressor Element 1
REST: RE1 silencing transcription factor
